# Model Prediction and Experimental Validation of Transverse Permeability of Large-Tow Carbon Fiber Composites

**DOI:** 10.3390/polym16091266

**Published:** 2024-05-01

**Authors:** Yu Feng, Qiaoxin Zhang, Jun Rao, Dong Liu

**Affiliations:** 1 School of Mechanical and Electronic Engineering, Wuhan University of Technology, Wuhan 430070, China; 15774549727@163.com (Y.F.); rao@whut.edu.cn (J.R.); 2 Zhongfu Shenying Carbon Fiber Co., Ltd., Lianyungang 222069, China; liudong312500@163.com

**Keywords:** large-tow carbon fiber, random distribution, stochastic model, transverse permeability

## Abstract

Large-tow carbon fiber (LCF) meets the low-cost requirements of modern industry. However, due to the large and dense number of monofilaments, there are problems with uneven and insufficient infiltration during material preparation. The permeability of large-tow carbon fibers can be used as a two-scale expression of resin flow during infiltration, making it an important factor to consider. This paper provides support for the study of pore formation. A two-dimensional model of randomly bundled large-filament carbon fibers is generated based on scanning electron microscope (SEM) maps. Microstructure size parameters are obtained, and a semi-analytical model of the transverse permeability of large-filament-bundled carbon fibers is established. Permeability values are then obtained. The analysis shows that the monofilaments in the tow are arranged randomly, and their periodic arrangement cannot be used to calculate permeability. Additionally, the number of monofilaments in a carbon fiber tow of the same volume fraction affects the permeability of the tow. Therefore, the permeability model of large-tow carbon fibers is reliable.

## 1. Introduction

### Model Development Based on Monofilament Gap

Carbon fiber has superior mechanical properties, including high specific strength and modulus. Carbon fiber composites are highly designable and are increasingly used in industries such as aerospace and the military [1,2,3,4,5]. However, the complex production process and high cost of raw materials greatly increase the manufacturing cost, making it challenging to widely use carbon fibers [6,7]. The cost of carbon fiber (CF) is a major contributor to the final cost of carbon fiber composites. Carbon fibers are mainly produced from polyacrylonitrile (PAN)-based precursors, which are oxidatively heat-stabilized and carbonized. These fibers are further treated by electronic surface treatment and coated with sizing agents. In this process, the cost of precursors is an important factor in the production of CFs. Large-tow carbon fiber is a cost-effective and efficient material that has rapidly developed, expanding the scope of application for carbon fiber composites. However, large-tow carbon fibers contain at least 24,000 monofilaments in a bundle [8]. This makes the fiber arrangement denser and the width of the unfolding of the tow wider, which poses a great challenge for the resin to infiltrate the tow. As a result, the resin cannot completely penetrate the large-tow carbon fibers, which inevitably leads to resin enrichment and porosity. This greatly reduces the strength of the large-tow carbon fiber composites [9,10,11,12]. The permeability of the fabric, defined as the ease with which resin flows through it, is used as an input to numerical resin flow simulations. This input is often used to inform the design of injection and exhaust ports, to avoid the formation of voids and dry spots.

The liquid composite molding process is a widely used method for manufacturing carbon fiber composites. The infiltration of carbon fibers and resin is primarily determined by the permeability of the reinforcement material. Therefore, the investigation of the permeability of carbon fibers in large tows is crucial for their subsequent molding process. Endruweit and Long [13] conducted a study on the impact of variations in the angle and spacing between fiber tows on the permeability of the fabrics. Bechtold and Ye [14] investigated the distribution of random filaments and determined the permeability of the fiber tows. Several studies have been conducted to determine the permeability of Darcy’s law by an ideal periodic carbon fiber arrangement [15,16,17,18,19].

Most studies on permeabilities have focused on the macroscopic scale along the filament bundle gap (millimeters) and the microscopic scale between filaments (micrometers) [20]. However, the dual scales have not been linked, as linking the microscale and macroscale complicates permeability calculations. The estimation of the filament bundle transverse permeability model remains the most dominant factor in all studies. Several studies [21,22,23,24] have investigated microscale models that involve filament beam impregnation and void formation leading to mesoscale cells, while others [25,26,27,28] have focused on the overall permeability characteristics. It is important to maintain a clear and logical structure when discussing technical topics. One study used a stochastic approach to investigate the effect of fiber distribution characteristics on micro-scale transverse permeability. Reference [29] aimed to establish a correlation between transverse permeability and average fiber spacing, considering a disordered fiber arrangement. Mayur G. Godbole [30] proposed a novel semi-analytical model for transverse filament permeability, using the flow assumptions of Gebart’s analysis [31]. This model was used to analyze the SEM of resin-injected filaments and develop a volume control method to investigate the effect of carbon fiber filament distribution on transverse permeability.

All of the studies mentioned above provide accurate estimates of the lateral permeability of filament bundles. Generally, this has a significant positive impact. This paper proposes a model for the transverse permeability of large-tow carbon fiber. The model takes into account the fact that the LCF tow is composed of multiple monofilaments stacked together. A stochastic two-dimensional model is established to account for the overlapping of the monofilaments in space. The microstructural dimensions are then incorporated into the newly constructed model to calculate the value of the large-tow carbon fiber permeability.

## 2. Theoretical Horizontal Permeability 

According to fluid dynamics, considering the resin as a Newtonian fluid and the carbon fiber bundle as a porous medium, the flow can be described by Darcy’s law, which is interpreted as the volumetric flow rate through a constant area (Q), is directly proportional to the cross-sectional area (A) and the pressure difference (∆p), and is inversely proportional to the viscosity of the fluid (μ) and to the length of the specimen (L).

(1) $$Q=K\frac{A}{\mu }\frac{∆p}{L}$$

**Figure 1:**
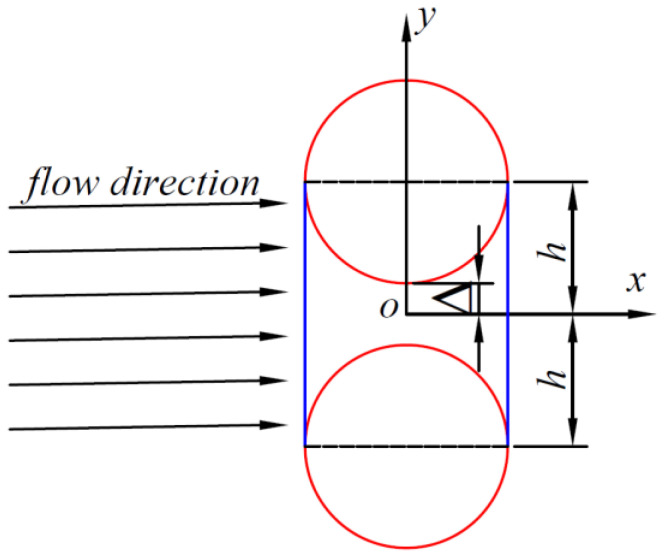
Special channel. The resin flow direction is perpendicular to the line of two carbon fibers. (The red lines represent carbon fiber, and the blue lines represent flow-through channels).

Considering the fiber bundle as a porous medium, the narrow space between the carbon fibers is viewed as a channel for resin flow, and the flow of resin in these channels can be described according to the equation of motion for the flow of an incompressible Newtonian fluid in a narrow space in fluid dynamics. If the pressure is guaranteed to be constant at both ends, and the pressure gradient is varied with the coordinates, then the velocity v(y) is varied by x, as shown below:

(2) $$v=\frac{h^{2}}{2\mu }\frac{\mathrm{dp}}{\mathrm{dx}}\left(\frac{y^{2}}{h^{2}}-1\right)$$

where h(x) is half the height of the channel, P is the pressure, μ is the viscosity, x is the flow direction, and y is the vertical flow direction. Figure 1 shows two carbon fiber filaments with the same diameter, and the blue area is the area of the channel flowing between the two carbon fibers, in which case the flow direction is perpendicular to the line between the two carbon fiber filaments, which is an extremely special case. In this case, we can use the Formula (2) to project when the resin is an incompressible Newtonian fluid flow between the two carbon fibers in the channel. Equation (2) demonstrates that the velocity of flow through the fiber channel in the x-direction is solely dependent on the y-coordinate. To determine the flow rate q per unit length of the carbon fiber channel (=Q_t_/L), it is necessary to integrate the channel cross-section (from –h to +h) concerning v. This can be expressed as $q=\int_{-h}^{h}vdy$.

By integrating Equation (2), it follows that

(3) $$q=-\frac{2h^{3}}{3\mu }\frac{\mathrm{dp}}{\mathrm{dx}}$$

From this, the pressure gradient is known:

(4) $$\frac{\mathrm{dp}}{\mathrm{dx}}=-\frac{3}{2}\frac{\mu q}{h^{3}\left(x\right)}$$

where the channel h between two carbon fibers can be found by the following equation

(5) $$h=\Delta +R(1-\sqrt{1-\frac{x^{2}}{R^{2}}})$$

This means

(6) $$h=\Delta +\frac{R}{2}\frac{x^{2}}{R^{2}}$$

Bringing Equation (6) into Equation (4) and integrating it, the calculation yields

(7) $$\Delta p=-\frac{3}{2}\frac{\mu q}{\Delta^{3}}\int_{a}^{b}{\left(\frac{1}{1+\frac{x^{2}}{2R\Delta }}\right)}^{3}\mathrm{dx}$$

It can be assumed that the following

(8) $$t=\frac{x}{\sqrt{2R\Delta }}$$

transferred to Equation (7), yields the following:

(9) $$\Delta p=-\frac{3}{2}\frac{\mu q}{\Delta^{3}}\sqrt{2R\Delta }\int_{t_{a}}^{t_{b}}{\left(\frac{1}{1+t^{2}}\right)}^{3}\mathrm{dt}$$

When the resin passes through the cross-section of two carbon fibers, x = R. From Darcy’s law, the magnitude of the permeability can be found as the following:

(10) $$K=\;-\frac{2\Delta^{3}}{3\sqrt{2R\Delta }}\frac{1}{f(R,\Delta )}$$

## 3. Carbon Fiber Large-Tow Transverse Permeability Modeling

**Figure 2:**
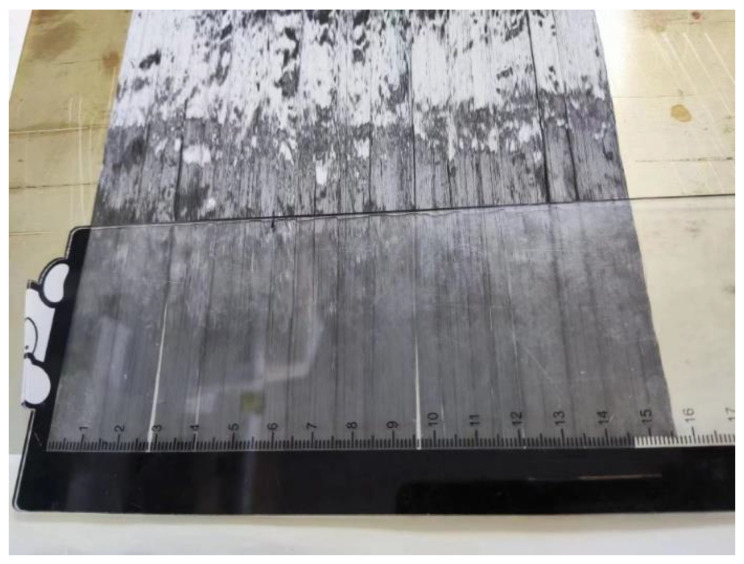
Zhongfu Shenying large-tow 48K carbon fiber tow.

As shown in Figure 2, this paper uses Zhongfu Shenying large-tow 48K carbon fiber tow with resin model EW800-1S. The single tow contains 48,000 monofilaments as shown in Figure 2, which creates great difficulty in resin penetration, whereas the number of carbon fiber filaments increases, which represents a smaller gap between the filaments.

### 3.1. Model Development Based on Monofilament Gap

**Figure 3:**
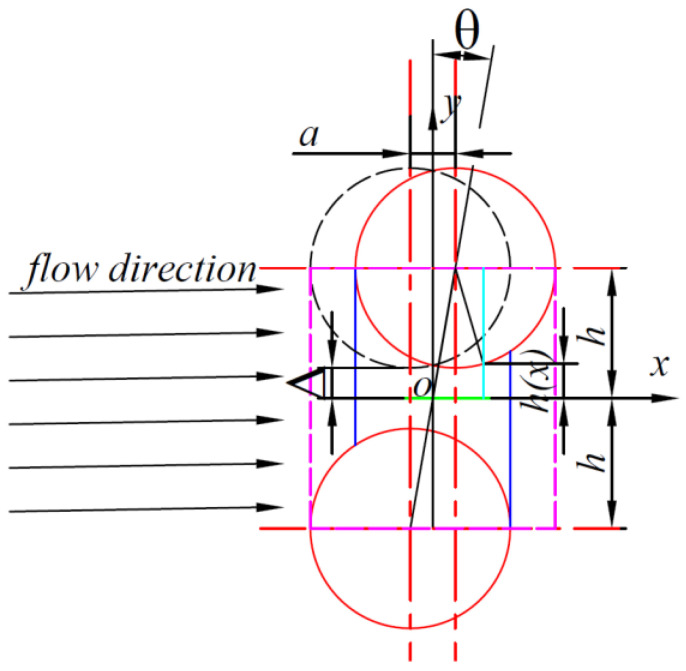
Carbon fiber offset type I channel. (The red solid line represents the carbon fiber, the blue solid line represents the flow-through channel, the black dashed line represents the special channel carbon fiber, and the purple dashed line represents the carbon fiber boundary).

When the two carbon fibers are offset, that is, when the distance between the two cylinders is not perpendicular to the flow direction, the flow of resin in the carbon fibers becomes unusually complex, and unlike the periodic distribution of carbon fibers with the same regularity, the flow of resin becomes chaotic, as shown in Figure 3.

Establish a coordinate system as shown in Figure 3. Assuming that the carbon fiber above is translated to the right in the *x*-direction by a distance a, and using the midpoint of the middle line as the origin of the coordinates, establish a Cartesian coordinate system as shown in Figure 2, and the height of the resin as it flows through the channel can be known:

(11) $$h_{1}\left(x\right)=\left(R+\Delta \right)-\sqrt{R^{2}-{\;\left(x-\frac{a}{2}\right)}^{2}}$$

For a carbon fiber pair with an offset central axis, the microstructural morphology dimensions are related to only three things: the center distance Δ between the two carbon fibers, the radius of the carbon fibers R, and the offset displacement a (offset angle θ). Therefore, a simplification of the above equation yields the following:

(12) $$h_{1}\left(x\right)=\Delta +\frac{R}{2}\frac{{\left(x-\frac{a}{2}\right)}^{2}}{R^{2}}$$

Bringing Equation (19) into the fluid equation yields the following:

(13) $$\frac{\mathrm{dp}}{\mathrm{dx}}=-\frac{3}{2}\frac{\mu q}{{\left(\Delta +\frac{R}{2}\frac{{\left(x-\frac{a}{2}\right)}^{2}}{R^{2}}\right)}^{3}}$$

Bringing Equation (19) into the fluid equation yields the following:

(14) $$\Delta p_{1}=-\frac{3}{2}\frac{\mu q}{\Delta^{3}}\int_{a}^{b}{\left(\frac{1}{1+\frac{{\left(x-\frac{a}{2}\right)}^{2}}{2R\Delta }}\right)}^{3}\mathrm{dx}$$

and

(15) $$t_{1}=\frac{x-\frac{a}{2}}{\sqrt{2R\Delta }}$$

Carried forward into Equation (21) above, the following is obtained:

(16) $$\Delta p_{1}=-\frac{3}{2}\frac{\mu q}{\Delta^{3}}\sqrt{2R\Delta }\int_{t_{a}}^{t_{b}}{\left(\frac{1}{1+{t_{1}}^{2}}\right)}^{3}\mathrm{dt}$$

The resin flows in the carbon fiber, regarded as an incompressible Newtonian fluid, and therefore the flow in the offset carbon fiber channel is a homogeneous flow with an integral range of the boundary of the offset carbon fiber, i.e., the integral range is the following:

(17) $$\Delta p_{1}=-\frac{3}{2}\frac{\mu q}{\Delta^{3}}\int_{-\left(R-\frac{a}{2}\right)}^{R-\frac{a}{2}}{\left(\frac{1}{1+\frac{{\left(x-\frac{a}{2}\right)}^{2}}{2R\Delta }}\right)}^{3}\mathrm{dx}$$

Given that x has the range of ($-R+\frac{a}{2},R-\frac{a}{2}$), the range of t1 can be deduced to be ($-\frac{R}{\sqrt{2R\Delta }},\frac{R-a}{\sqrt{2R\Delta }}$), where f1 can take values ranging from 0. When its value is positive, one of the carbon fibers above is offset to the right; when its value is negative, the carbon fiber above is offset to the left. When its absolute value is greater than or equal to R, the two carbon fibers do not have a channel through which the resin flows. However, the pressure of its passage is still affected by the change of its value and increases with the increase of distance.

The analysis revealed that

(18) a=2(R+Δ)tanθ

Then,

(19) $$\Delta p_{1}=-\frac{3}{2}\frac{\mu q}{\Delta^{3}}\sqrt{2R\Delta }\int_{-\frac{R}{\sqrt{2R\Delta }}}^{\frac{R-a}{\sqrt{2R\Delta }}}{\left(\frac{1}{1+{t_{1}}^{2}}\right)}^{3}\mathrm{dt}$$

Make

(20) $$f_{1}\left(R,\Delta ,a\right)=\int_{-\frac{R}{\sqrt{2R\Delta }}}^{\frac{R-a}{\sqrt{2R\Delta }}}{\left(\frac{1}{1+{t_{1}}^{2}}\right)}^{3}\mathrm{dt}$$

Therefore:

(21) $$\Delta p_{1}=-\frac{3}{2}\frac{\mu q}{\Delta^{3}}\sqrt{2R\Delta }\cdot f_{1}\left(R,\Delta ,a\right)$$

From Darcy’s law, the magnitude of permeability can be found as

(22) $$K_{1}=\frac{2\Delta^{3}}{3\sqrt{2R\Delta }}\frac{1}{f_{1}\left(R,\Delta ,a\right)}$$

The above equation shows that the permeability of the two carbon fiber channels is related to the size, spacing, and offset of the carbon fibers.

**Figure 4:**
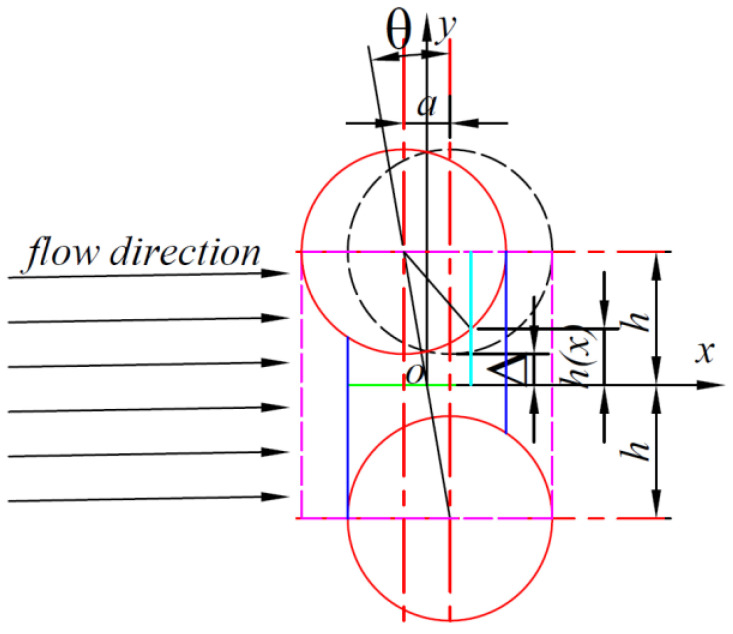
Carbon fiber offset type II channel. (The red solid line represents the carbon fiber, the blue solid line represents the flow-through channel, the black dashed line represents the special channel carbon fiber, and the purple dashed line represents the carbon fiber boundary).

Similarly, by establishing a coordinate system as shown in Figure 4, assuming that the carbon fiber above is translated to the left in the *x*-direction by a distance a, and using the midpoint of the middle line as the origin of the coordinates to establish a Cartesian coordinate system as shown in Figure 4, the height of the resin as it flows through the channel can be known:

(23) $$h_{2}\left(x\right)=\left(R+\Delta \right)-\sqrt{R^{2}-{\left(x+\frac{a}{2}\right)}^{2}}$$

From the fact that x has the range ($-R+\frac{a}{2},R-\frac{a}{2}$), it follows that t2 has the range ($-\frac{R-a}{\sqrt{2R\Delta }},\frac{R}{\sqrt{2R\Delta }}$). It then yields the following:

(24) $$\Delta p_{2}=-\frac{3}{2}\frac{\mu q}{\Delta^{3}}\sqrt{2R\Delta }\int_{-\frac{R-a}{\sqrt{2R\Delta }}}^{\frac{R}{\sqrt{2R\Delta }}}{\left(\frac{1}{1+{t_{2}}^{2}}\right)}^{3}\mathrm{dt}$$

Make

(25) $$f_{2}\left(R,\Delta ,a\right)=\int_{\frac{a-R}{\sqrt{2R\Delta }}}^{\frac{R}{\sqrt{2R\Delta }}}{\left(\frac{1}{1+{t_{2}}^{2}}\right)}^{3}\mathrm{dt}$$

Therefore:

(26) $$\Delta p_{2}=-\frac{3}{2}\frac{\mu q}{\Delta^{3}}\sqrt{2R\Delta }\cdot f_{2}\left(R,\Delta ,a\right)$$

From Darcy’s law, the magnitude of permeability can be found as

(27) $$K_{2}=\frac{2\Delta^{3}}{3\sqrt{2R\Delta }}\frac{1}{f_{2}\left(R,\Delta ,a\right)}$$

When estimating the permeability of the large tow, it is important to consider multiple carbon fibers. The resin flow rate through the carbon fiber of the large tow is equal to the sum of the flow rates through each of the active channels.

(28) $$Q_{T}=\sum_{i=1}^{N}q_{i}$$

The flow between two carbon fibers per localization is represented by q_i_.

(29) $$q_{i}=K_{i}\frac{A_{i}}{\mu }\frac{∆p_{i}}{L_{i}}$$

An infinite number of microscopic channels are formed between every two adjacent carbon fibers, and the sum of the flows in all these channels at a given moment constitutes the total flow through the carbon fibers.

The total flow rate through the entire area of the selected portion of the carbon fiber tow can be expressed by the following equation:

(30) $$Q_{T}=NK_{T}\frac{A_{\mathrm{tow}}}{\mu }\frac{∆p}{L_{\mathrm{tow}}}$$

By combining the two equations mentioned above, it is possible to derive the total permeability of the filament bundle.

(31) $$K_{T}=\frac{1}{N}\sum_{i=1}^{N}K_{i}\left(\frac{d_{i}}{H^{\prime }}\right)\left(\frac{L_{\mathrm{tow}}}{2R-a}\right)\frac{∆p_{i}}{∆p}$$

To obtain the total permeability of the filament bundle from the equation above, it is necessary to know all parameter values. The variable N in the equation represents the number of channels in the filament bundle, which can be determined through numerical analysis, along with the corresponding permeability of each microscopic channel. K_i_ can also be calculated using the previous calculations. The shape parameters of the microchannels and microchannels can be deduced from the previous chapters. The only parameter that is not easily derived is the microscopic local pressure, ∆pi/∆p, which can be calculated using the Hagen–Poiseuille formulae for the microscopic and macroscopic channels.

(32) $$∆p_{i}=\frac{C\mu \bar{u_{i}}L_{i}}{d_{i}^{2}}$$

(33) $$∆p=\frac{C\mu \overset{-}{u}L_{\mathrm{tow}}}{H^{\prime 2}}$$

where C is the shape factor, which is a constant; $\overset{-}{u}$ is the thickness of the resin flowing through the filament bundle at a given moment; $\bar{u_{i}}$ is the diameter of the carbon fiber filament flowing through the resin at a given moment; $L_{i}=2R-a$; $d_{i}=2\left(R+\Delta \right)-\sqrt{\left(2R-a\right)a}$.

The pressure ratio can be obtained:

(34) $$\frac{∆p_{i}}{∆p}=\frac{R\left(2R-a\right)}{d_{i}^{2}}\frac{H^{\prime }}{L_{\mathrm{tow}}}$$

The insertion of Equation (34) into Equation (31) gives the following:

(35) $$K_{T}=\frac{1}{N}\sum_{i=1}^{N}(K_{i}\frac{R}{d_{i}})=\frac{1}{N}\sum_{i=1}^{N}(\frac{2\Delta^{3}}{3\sqrt{2R\Delta }}\frac{R}{2\left(R+\Delta \right)-\sqrt{\left(2R-a\right)a}}\frac{1}{f_{i}\left(R,\Delta ,a\right)})$$

From the above equation, it can be seen that $K_{T}$ is a positive number; then, the following must be required: 0<a<2R.

Up to this point, the permeability of the final large-tow carbon fiber is only related to the values of the micro-parameters of all the micro-channels of the whole tow. From the above equation, it can be seen that if all values of R, Δ, and a are obtained, the process is computationally huge and does not truly reflect the permeability of the resin. Therefore, in this paper, we will use computer simulation to simulate the distribution of carbon fibers in the 48K macro-bundle carbon fiber composites.

### 3.2. Large Tow Stochastic Model

**Figure 5:**
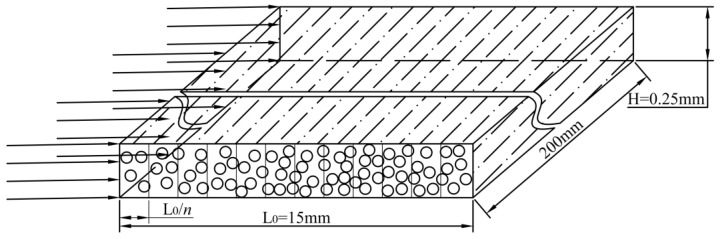
A three-dimensional representation of a ribbon. (The arrows represent the direction of resin flow, the circles represent the carbon fibers, and the dotted lines represent the distribution of the carbon fibers in the ribbon).

The test results indicate that the large-stranded carbon fiber tow tape has a width of 15 mm and a thickness of 0.25 mm. A length of 200 mm was used for the test, as shown in Figure 5.

**Figure 6:**
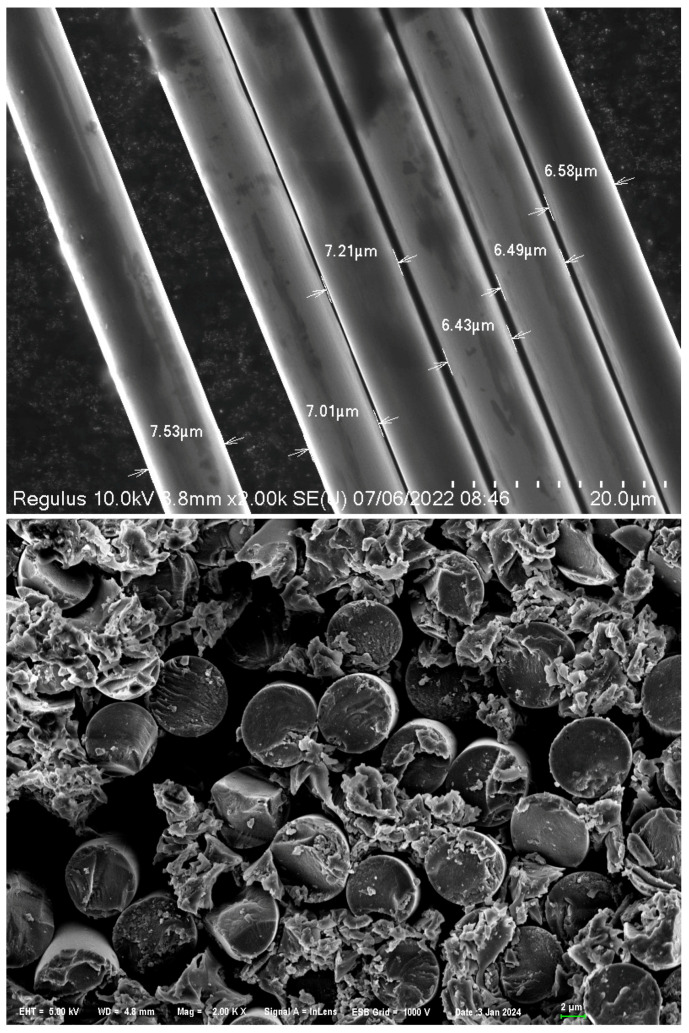
Diameter measurement of large-tow carbon fibers. (Arrows indicate carbon fiber diameter).

The monofilament diameter of 48K large-tow carbon fibers was measured using SEM, as shown in Figure 6. The average monofilament diameter was found to be 7 μm. Assuming that all carbon fiber monofilaments are circular and have the same diameter, the cross-section of a large-tow carbon fiber can be approximated as a rectangle with dimensions of 15 mm by 0.25 mm. This rectangle contains 48,000 circles with a diameter of 0.007 mm each.

**Figure 7:**
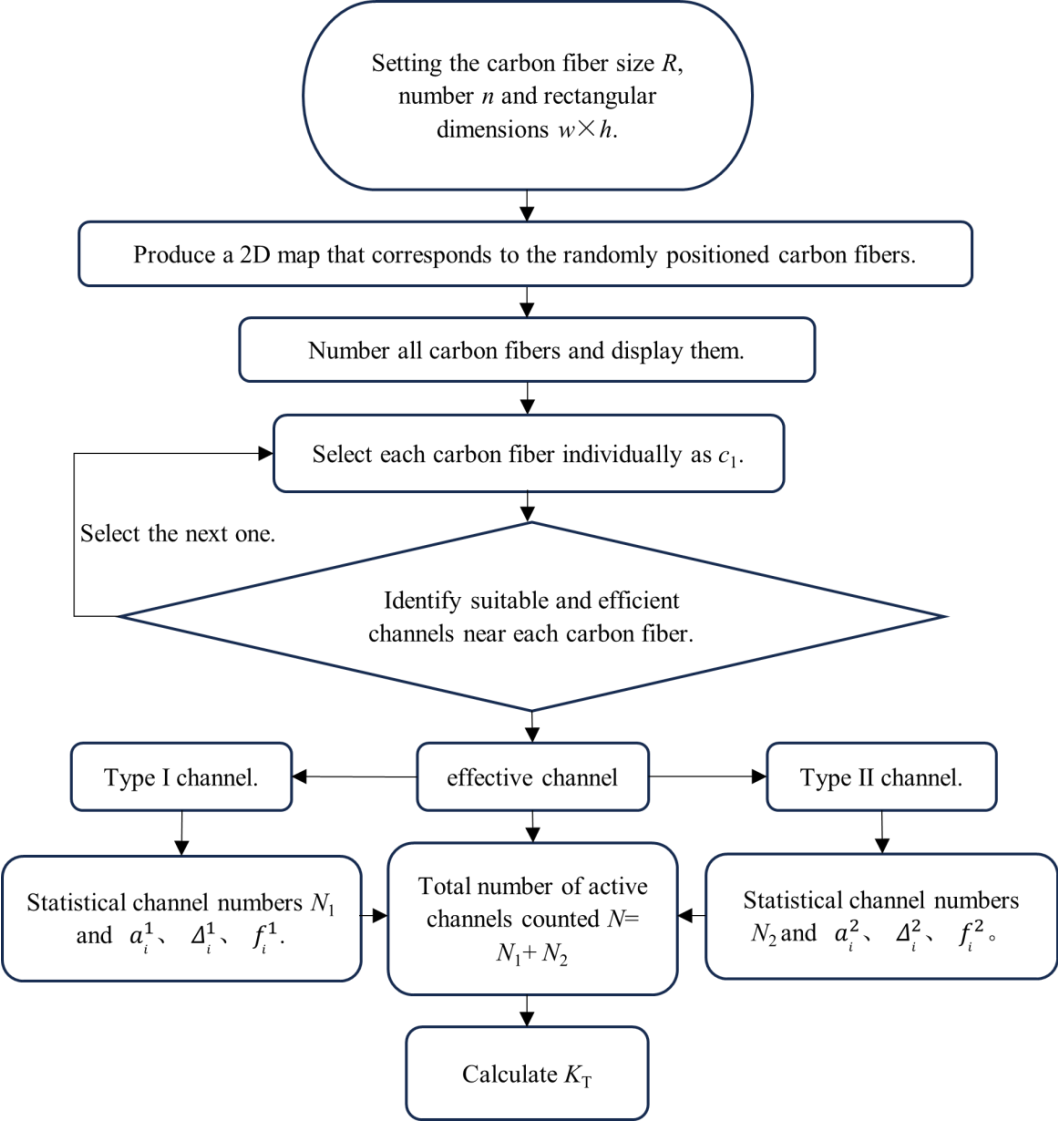
The process of computer simulation.

The algorithm requires the placement of 48,000 circles with a diameter of 7 μm in a rectangle with a length of 15,000 μm and a width of 250 μm to construct the cross-section. This paper assumes a uniform distribution of carbon fibers in the cross-section of a large-tow carbon fiber, with a width much larger than the thickness. The width direction is uniformly divided into n points. For the case where n = 1000, the task involves placing 48 circles with a diameter of 7 μm in a rectangle with a length of 15 μm and a width of 250 μm. Similarly, for n = 100, the task involves placing 480 circles of 7 μm diameter in a rectangle of length 150 μm and width 250 μm. For n = 10, the task involves placing 48 circles of 7 μm diameter in a rectangle of length 1500 μm and width 250 μm. Finally, for n = 1, the task involves placing 48 circles of 7 μm diameter in a rectangle of length 15,000 μm and width 250 μm. The algorithm’s flow is illustrated in Figure 7.

**Figure 8:**
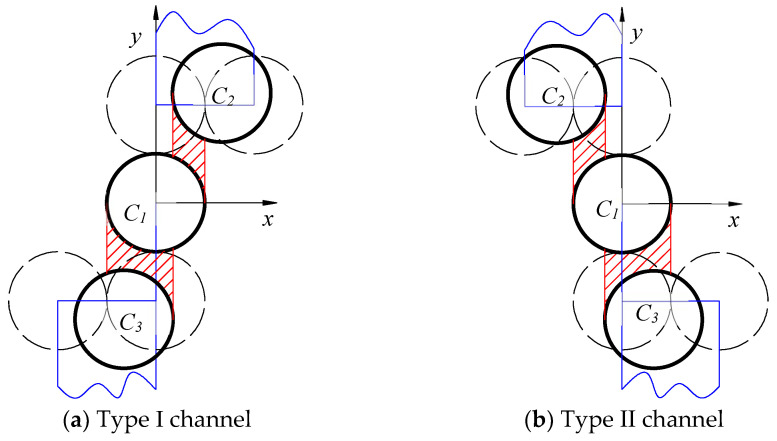
Evaluation of the effectiveness of channels in large filament bundles. (**a**) $-2R<x_{1}-x_{2}\leq 0$ and $-4R<y_{1}-y_{2}<-2R$; (**b**) $0\leq x_{1}-x_{2}<2R$ and $-4R<y_{1}-y_{2}<-2R$. (The red area represents the resin flow through the channel, while the blue area represents the position of the center of the circle of C2 and C3 that meets the specified requirements).

From the above computer simulation, the position of the center of all simulated carbon fiber circles can be calculated, so that the center of the circle that meets the requirements can be obtained by judging the position of each center. In this paper, the channel formed by two carbon fibers is divided into the type I channel and the type II channel, as shown in Figure 8. In certain instances, C2 or C3 may be included in both type I and type II channels. This is the rationale behind the distinction between type I and type II channels. It should be noted that the presence of a particular fiber in both a type I and a type II channel does not necessarily imply that the channel itself is of both types.

Firstly, all carbon fibers are numbered. Then, the carbon fibers around each fiber are evaluated. Type I channels are evaluated and paired, followed by counting all the type I channels. Next, type II channels are evaluated and paired, followed by counting all the type II channels. The coordinates of all circles are extracted based on the randomly generated circle coordinates above. They are then numbered and evaluated around each carbon fiber to determine the number of type I and type II channels. The counts are then accumulated.

**Figure 9:**
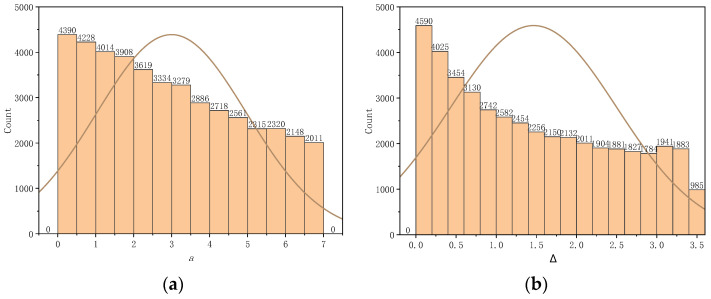
Computer simulation of large tow carbon fibers to obtain distributions of the parameters a and Δ. (**a**) The parameter a is within the range of 0 to 7 μm. (**b**) The parameter Δ is within the range of 0 to 3.6 μm. (The curve depicted in the figure represents a probability distribution curve).

The distribution of carbon fibers was simulated by a computer, and the parameters of the effective channels were obtained by calculating the simulated carbon fibers. Figure 9 displays the distribution of a and Δ. The overall transverse permeability was obtained using the formula for permeability shown in Table 1. The permeability of the large filament bundle was 1.8004 × 10^−12^ m^2^.

## 4. Experimental Section

**Figure 10:**
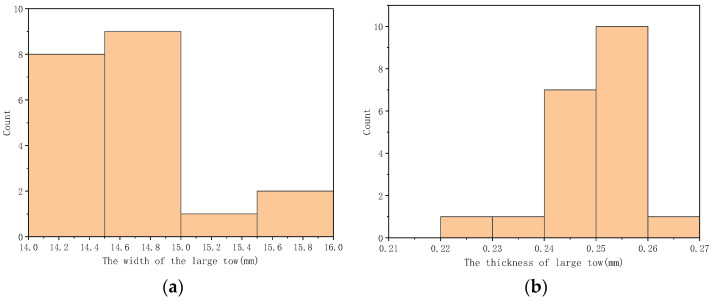
The statistical distribution of measurements for the width and thickness of large filament tows. (**a**) The distribution of the width. (**b**) The distribution of the thickness.

Zhongfu Shenying selected a large 48K carbon fiber tow, using the resin model EW800-1S. The monofilament tow contained 48,000 monofilaments, and different segments of the carbon fiber tow were selected from one spool. The width and thickness of the ribbon were obtained from the measurement of the monofilament tows, which were 15 mm and 0.25 mm, respectively. The distribution of the width and thickness of the ribbons was measured and counted, as shown in Figure 10.

**Figure 11:**
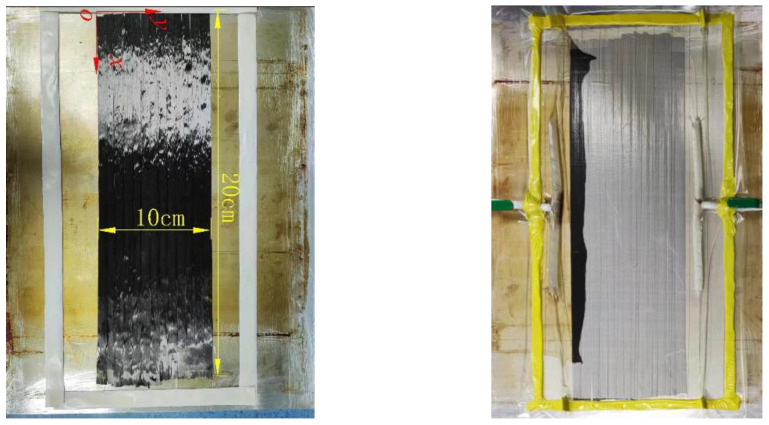
Experiment on measuring the permeability of large-tow carbon fibers.

The carbon fiber tow tape was intercepted and laid flat on a smooth aluminum plate, aligning the carbon fiber tows to form a rectangular shape measuring 20 cm in length and 10 cm in width, as depicted in Figure 11. Subsequently, double-sided adhesive tape was applied around the rectangular ribbon, and a layer of gauze was added to enable clear observation of the resin flow in the carbon fiber. In this instance, the flexible vacuum bag is employed as the upper mold, with the vacuum bag fully enclosing the large bundle of carbon fibers. This ensures that there is no leakage of the experimental vacuum bag and that no air bubbles are generated during the experiment. This, in turn, ensures that the resin is carried out in a completely negative pressure, thus improving the credibility of the experiment.

The carbon fiber filament tape should have the spiral hose placed at both ends, with one end serving as the resin fluid inlet and the other as the vacuum port. The spiral hose should be wrapped around the fluid valve, which can be controlled by a switch. Finally, a vacuum bag should be laid and completely covered with double-sided tape to ensure that the entire test device is sealed within the vacuum bag, preventing any pressure leakage during vacuuming. The experiment involved immersing one end of the fluid control valve in resin and connecting the other end to the vacuum pump. The vacuum pump was then started to experiment with the resin lateral infiltration of the large-tow carbon fibers along the *y*-axis. The position and time of resin flow through the large-tow carbon fibers were observed and measured in real time.

Therefore, Darcy’s law can be used to deduce the permeability of the resin that infiltrates large-tow carbon fibers.

(36) $$k=\frac{\phi \mu h^{2}}{2\mathrm{tP}}$$

The permeability to be predicted is denoted by k, while the porosity is denoted by ϕ, the fluid viscosity by μ, the length of the filament bundle infiltrated by the resin by h, the infiltration time by t, and the pressure difference by P.

Table 2 displays the results of measuring negative pressure as the resin flowed into the large-tow carbon fibers at distances of 10 mm, 15 mm, 18 mm, and 20 mm away from the inlet position. The elapsed time for each distance was 330 s, 520 s, 650 s, and 700 s, respectively. The tracing point method indicates that the resin flow through time is essentially linear, except for the final point, which is slightly shorter. During the experiment, it was observed that following the resin’s flow through the extensive bundle of carbon fibers for a specific distance, the area surrounding the measuring plate devoid of carbon fibers was filled with resin, and the resin flowed into the carbon fiber bundle through the side path. This resulted in a shorter time at the rear. The corresponding permeability values were calculated for each position. By comparing the data, it was found that the permeability values were closer to the numerical solution when the distance was greater. The experimental permeability results may contain errors due to the resin inlet location, vacuum bag installation, resin flow path, and the location of the large-bundle carbon fiber arrangement in the experiment. The experimental uncertainties are difficult to eliminate. The mean value of the experimental results is 1.8632 × 10^−12^ mm^2^, and the standard deviation is 4.70512 × 10^−13^ mm^2^. These values indicate that all the results are within one order of magnitude and meet the experimental requirements, making them acceptable.

## 5. Conclusions

This paper proposes a new transverse permeability model for 48,000 large-tow carbon fibers. To account for a large number of monofilaments and the small gaps between them, a computer simulation was used to develop a microscopic effective channel model and extract all the necessary parameters, including ‘a’ and ‘Δ’. The specific values are obtained by incorporating the parameters above into the large-tow carbon fiber transverse permeability model. The model was verified by comparing the simulation results with the experimental results. The following conclusions can be drawn:

The arrangement of monofilaments in the tow is random, making it impossible to calculate the permeability by periodic arrangement.

The number of monofilaments in the tow of carbon fiber with the same volume fraction affects the permeability of the tow.

The method employs a calculation parameterized by the number of fibers and fiber microstructure, without restricting the density and volume of carbon fibers in large tows. Consequently, the method applies to the estimation of transverse permeability for different tow densities and tow volumes.

The predicted permeability value for 48,000 large-tow carbon fibers is 2.0971 × 10^−12^ mm^2^, which is close to the experimentally measured value of 1.8632 × 10^−12^ mm^2^. It is important to note that the language used in this conclusion is clear, objective, and value-neutral, adhering to the desired characteristics outlined in the assignment.

## Figures and Tables

**Table 1:** The permeability was obtained through simulation for varying numbers of fibers.

Number of Carbon Fibers	Number of Type I Channels	Number of Type II Channels	Active Channels Count	Horizontal Permeability
48 (15 μm × 250 μm)	29	29	58	3.3745 × 10^−12^
480 (150 μm × 250 μm)	321	325	646	2.5912 × 10^−12^
4800 (1500 μm × 250 μm)	2097	3141	6238	2.5002 × 10^−12^
48,000 (15,000 μm × 250 μm)	31,611	31,624	63,235	2.0971 × 10^−12^

**Table 2:** Permeability changes obtained through experimentation.

Trial No.	Inlet Length, *h* (mm)	Inlet Time, *t* (s)	Viscosity, *μ* (cp)	Porosity, *ϕ*	Pressure Difference, *P* (Pa)	Permeability, (m^2^)
1	10	330	1040	0.508	64,000	$1.2508\times 10^{-12}$
2	15	520	1040	0.508	64,000	$1.7859\times 10^{-12}$
3	18	650	1040	0.508	64,000	$2.0574\times 10^{-12}$
4	20	700	1040	0.508	64,000	$2.3586\times 10^{-12}$

## Data Availability

Data are contained within the article.

## References

[1] Dai D., Lan X., Wang Z. (2023). Hierarchical carbon fiber reinforced SiC/C aerogels with efficient electromagnetic wave absorption properties. Compos. Part B Eng..

[2] Hong H., Bae K.J., Jung H., Oh Y., You N.-H., Lee J.-C., Yu J. (2022). Preparation and characterization of carbon fiber reinforced plastics (CFRPs) incorporating through-plane-stitched carbon fibers. Compos. Struct..

[3] Qu C.-B., Wu T., Huang G.-W., Li N., Li M., Ma J.-L., Liu Y., Xiao H.-M. (2021). Improving cryogenic mechanical properties of carbon fiber reinforced composites based on epoxy resin toughened by hydroxyl-terminated polyurethane. Compos. Part B Eng..

[4] Yao T.-T., Zhang X.-F., Zhang W.-S., Liu Y.-T., Liu Q., Wu G.-P. (2021). Controlled attachment of polycarbonate nanoparticles on carbon fibers for increased resin impregnation and interfacial adhesion in carbon fiber composites. Compos. Part B Eng..

[5] Shirolkar N., Maffe A., DiLoreto E., Gulgunje P., Gupta K., Park J.G., Kirmani M.H., Liang R., Kumar S. (2023). Continuous small diameter carbon fibers. Carbon.

[6] Khan H., Kaur J., Naebe M., Hutchinson S., Varley R.J. (2022). Continuous, pilot-scale production of carbon fiber from a textile grade PAN polymer. Mater. Today Commun..

[7] Hiremath N., Young S., Ghossein H., Penumadu D., Vaidya U., Theodore M. (2020). Low cost textile-grade carbon-fiber epoxy com-posites for automotive and wind energy applications. Compos. Part B Eng..

[8] Song Y., Liu C., Li H., Xu K., Geng H., Wu H., Zu L., Jia X., Ge L., Yang X. (2023). Optimizing dual-scale wettability of epoxy resin on large-tow carbon fiber via tension-driven capillary wicking. Compos. Part B Eng..

[9] Sun T., Zhang X., Qiu B., Luo Y., Ling Y., Chen Y., Xu Z., Liang M., Zou H. (2022). Controllable construction of gradient modulus intermediate layer on high strength and high modulus carbon fibers to enhance interfacial properties of epoxy composites by efficient electrochemical grafting. Compos. Part B Eng..

[10] Kumar B.S., Chandrakant J.S., Di B.Y. (2019). Experimental and microscopic investigation on mechanical performance of textile spread-tow thin ply composites. Fibers Polym..

[11] Wang J., Anthony D.B., Fuentes C.A., De Luca H.G., Zhang D., Bismarck A., Van Vuure A.W., Shaffer M.S., Seveno D. (2022). Wettability of carbon nanotube-grafted carbon fibers and their interfacial properties in polypropylene thermoplastic composite. Compos. Part A Appl. Sci. Manuf..

[12] Lim S.H., On S.Y., Kim H., Bang Y.H., Kim S.S. (2021). Resin impregnation and interfacial adhesion behaviors in carbon fiber/epoxy composites: Effects of polymer slip and normalized surface free energy with respect to the sizing agents. Compos. Part A Appl. Sci. Manuf..

[13] Endruweit A., Long A.C. (2006). Influence of stochastic variations in the fibre spacing on the permeability of bi-directional textile fabrics. Compos. Part A Appl. Sci. Manuf..

[14] Bechtold G., Ye L. (2003). Influence of fibre distribution on the transverse flow permeability in fibre bundles. Compos. Sci. Technol..

[15] Sangani A.S., Yao C. (1988). Transport processes in random arrays of cylinders. II. Viscous flow. Phys. Fluids.

[16] Cai Z., Berdichevsky A.L. (1993). Numerical simulation on the permeability variations of a fiber assembly. Polym. Compos..

[17] Lundström T.S., Gebart B.R. (1995). Effect of perturbation of fiber architecture on permeability inside fiber tows. J. Compos. Mater..

[18] Chen X., Papathanasiou T. (2007). Micro-scale modeling of axial flow through unidirectional disordered fiber arrays. Compos. Sci. Technol..

[19] Chen X., Papathanasiou T.D. (2008). The transverse permeability of disordered fiber arrays: A statistical correlation in terms of the mean nearest interfiber spacing. Transp. Porous Media.

[20] Huan T., Pillai K.M. (2010). Fast liquid composite molding simulation of unsaturated flow in dual-scale fiber mats using the imbibition characteristics of a fabric-based unit cell. Polym. Compos..

[21] Parnas R.S., Salem A.J., Sadiq T.A., Wang H.-P., Advani S.G. (1994). The interaction between micro- and macro-scopic flow in RTM preforms. Compos. Struct..

[22] Binétruy C., Hilaire B., Pabiot J. (1997). The interactions between flows occurring inside and outside fabric tows during rtm. Compos. Sci. Technol..

[23] Binetruy C., Hilaire B., Pabiot J. (1998). Tow impregnation model and void formation mechanisms during RTM. J. Compos. Mater..

[24] Chan A.W., Morgan R.J. (1993). Tow impregnation during resin transfer molding of bi-directional nonwoven fabrics. Polym. Compos..

[25] Pillai K.M., Advani S.G. (1998). Numerical simulation of unsaturated flow in woven fiber preforms during the resin transfer molding process. Polym. Compos..

[26] Pillai K.M., Advani S.G. (1998). A model for unsaturated flow in woven fiber preforms during mold filling in resin transfer molding. J. Compos. Mater..

[27] Simacek P., Advani S.G. (2003). A numerical model to predict fiber tow saturation during liquid composite molding. Compos. Sci. Technol..

[28] Wang Y., Grove S. (2008). Modelling microscopic flow in woven fabric reinforcements and its application in dual-scale resin infusion modelling. Compos. Part A Appl. Sci. Manuf..

[29] Endruweit A., Gommer F., Long A. (2013). Stochastic analysis of fibre volume fraction and permeability in fibre bundles with random filament arrangement. Compos. Part A Appl. Sci. Manuf..

[30] Godbole M.G., Purandare R., Harshe R., Hood A., Gururaja S., Joshi M., Advani S. (2019). Influence of filament distribution on transverse tow permeability: Model predictions and experimental validation. Compos. Part A Appl. Sci. Manuf..

[31] Gebart B. (1992). Permeability of Unidirectional Reinforcements for RTM. J. Compos. Mater..

